# Effect of histamine H3 receptor selective agonist imetit on cough and symptoms of allergic rhinitis in animal model of upper airway cough syndrome

**DOI:** 10.1186/2045-7022-5-S4-P13

**Published:** 2015-06-26

**Authors:** Eva Kovacova, Silvia Gavliakova, Tomas Buday, Jana Plevkova

**Affiliations:** 1 Jessenius Faculty of Medicine, Comenius University, Department of Pathophysiology, Martin, Slovakia

## Background

Upper airway diseases (e.g. allergic rhinitis (AR)) are one of most common causes of chronic cough in subjects with negative X-ray finding. In UACS (upper airway cough syndrome), cough is strongly associated with the ongoing nasal inflammation. Effective treatment of nasal inflammation may lead to down regulation of pathologically enhanced chronic cough. Histamine plays a critical role in upper airway diseases. All known histamine receptors (H1R, H2R, H3R, and H4R) have been demonstrated in the nasal mucosa. Old generation of antihistamines (inverse agonists of H1R) are empirically used in treatment of UACS, although their clinical use is limited by their serious adverse effects. The aim of our study was to ascertain the effect of H3R selective agonist imetit - known to suppress release of substance P from afferent nerves in allergic nasal inflammation on cough and symptoms of AR in an animal model.

## Method

Guinea pigs (n=10) were sensitised by i.p. administration of ovalbumin (OVA). Sensitisation was confirmed 21 days later by skin prick tests. Animals were repeatedly challenged with i.n. OVA to induce AR and enhanced cough reflex. When the reliability of the model was confirmed, animals were pretreated by i.p. administration of imetit dihydrobromide (1mg/kg and 2mg/kg of body weight) 30 min. prior i.n. OVA administration. AR was evaluated from the occurrence of typical clinical symptoms including sneezing, conjunctival and nasal secretion, or nasal acoustic phenomenon. The effect on cough was assessed from the response to inhalation of citric acid (0.4M, 10 min) in double chamber plethysmograph. Final cough count and cough latency were analysed from the airflow traces, presence of cough motor pattern and the cough sound analysis.

## Results

AR up-regulated the cough response from 9±2 to 16±1 cough per provocation, med±IQR, p<0.05 and shortened cough latency. Imetit (1mg/kg) suppressed nasal symptoms and decreased number of cough from 16±1 to 12±1, however the data did not reach significance. Imetit (2mg/kg) significantly suppressed the nasal symptoms, and number of coughs from 16±1 to 6±2, med±IQR, p<0.05. Imetit in both doses did not significantly influence cough latency.

## Conclusion

Imetit in both doses reduces the symptoms of AR, however only the dose 2mg/kg reduces significantly total cough count. Cough latency was not significantly influenced by imetit.

